# The bacterial DNA repair protein Mfd confers resistance to the host nitrogen immune response

**DOI:** 10.1038/srep29349

**Published:** 2016-07-20

**Authors:** Elisabeth Guillemet, Alain Leréec, Seav-Ly Tran, Corinne Royer, Isabelle Barbosa, Philippe Sansonetti, Didier Lereclus, Nalini Ramarao

**Affiliations:** 1 Micalis Institute, INRA, AgroParisTech, Université Paris-Saclay, 78350 Jouy-en-Josas, France; 2 INRA, UMR 203, 11 Av J. Capelle, 69621 Villeurbanne, France; 3 INSERM U786, Institut Pasteur, 28 rue du Dr Roux, 75724 Paris Cedex 15, France

## Abstract

Production of reactive nitrogen species (NO) is a key step in the immune response following infections. NO induces lesions to bacterial DNA, thus limiting bacterial growth within hosts. Using two pathogenic bacteria, *Bacillus cereus* and *Shigella flexneri*, we show that the DNA-repair protein Mfd (Mutation-Frequency-Decline) is required for bacterial resistance to the host-NO-response. In both species, a mutant deficient for *mfd* does not survive to NO, produced *in vitro* or by phagocytic cells. *In vivo*, the ∆*mfd* mutant is avirulent and unable to survive the NO-stress. Moreover, NO induces DNA-double-strand-breaks and point mutations in the Δ*mfd* mutant. In overall, these observations demonstrate that NO damages bacterial DNA and that Mfd is required to maintain bacterial genomic integrity. This unexpected discovery reveals that Mfd, a typical housekeeping gene, turns out to be a true virulence factor allowing survival and growth of the pathogen in its host, due to its capacity to protect the bacterium against NO, a key molecule of the innate immune defense. As Mfd is widely conserved in the bacterial kingdom, these data highlight a mechanism that may be used by a large spectrum of bacteria to overcome the host immune response and especially the mutagenic properties of NO.

The host defense against bacteria is predominantly mediated by cellular immune mechanisms. Phagocytic neutrophils and macrophages infiltrate inflamed areas, where they produce an extensive array of toxic substances. The chemicals secreted include reactive oxygen and nitrogen species, which cause DNA damage and gene mutations^1^,^2^. Nitric oxide (NO) is synthesized by an enzymatic reaction involving NO synthases (NOS). NOS are expressed both as constitutive enzymes, which contribute to vasorelaxation and neurotransmission, and as inducible isoforms (iNOS). Various cells including macrophages, neutrophils and epithelial cells express iNOS, and an excess of NO is produced during most types of infections. NO can damage biological molecules including proteins and nucleic acids^3^. NO is cytotoxic and mutagenic both for various pathogens and for host cells^2^,^4^,^5^. Thus, NO plays an important and complex role during infections, limiting microbial proliferation within host cells and contributing to microbial clearance.

Bacteria express sensor proteins able to detect NO, and switch on the expression of enzymes that detoxify NO before it reaches lethal levels^6^. In *E. coli*, a mutant deficient for the nucleotide excision repair (NER) pathway is sensitive to HNO_2_ treatment^7^ and the base excision repair (BER) pathway protects *Salmonella* from the genotoxic effects of the host NO^8^. RecBCD-dependent recombinational repair also plays a role in preventing the genotoxic effects of NO. The NO sensitivity in the absence of RecBCD-dependent homologous recombination indicates that NO toxicity is due, at least partially, to the formation of DNA double-strand breaks (DSBs)^9^.

Damage to DNA can affect transcription fidelity and processivity and thereby threatens cell viability. DNA lesions that block RNA polymerase (RNAP) prevent transcription. In bacteria, RNAP stalling triggers a specialized DNA repair mechanism, called transcription coupled repair (TCR) pathway. Mfd (Mutation Frequency Decline) is an evolutionarily conserved bacterial protein involved in TCR^10^. Mfd removes RNAP stalled by DNA damage. Mfd utilizes ATP to translocate along DNA, most likely forcing RNAP forward and ultimately dissociating it from the DNA template^10^. In *E. coli*, Mfd also contains binding domains that may recruit UvrA and trigger the associated NER pathway^11^. It was recently shown in *B. subtilis* that Mfd and UvrA act in the same pathway in the context of transcription^12^. Mfd also decreases the efficiency with which RNAP bypasses abasic sites, possibly reducing the level of transcriptional mutagenesis caused by these DNA lesions^13^. Mfd is required for DNA repair in *E. coli* following experimental UV irradiation^14^ and in *Helicobacter pylori* following exposure to mitomycin C^15^. Mfd was also found to be important for processing the genetic damage during *Bacillus subtilis* stationary phase associated mutagenesis^16^ and sporulation^17^,^18^ and during DNA recombination^19^. Furthermore, Mfd has been associated with the development of antibiotic resistance in *Campylobacter jejuni* and *H. pylori*^15^,^20^. Thus, the mechanism of adaptive mutagenesis and DNA repair by Mfd has been thoroughly studied in the context of both artificially induced and spontaneous mutations^14^,^16^,^21^,^22^. However, to our knowledge its involvement in bacterial pathogenesis has never been reported.

In this study, we show that the bacterial Mfd protein is essential to survive the deleterious effect of the nitrogen response. We use two different human pathogens, the Gram-positive *Bacillus cereus* and the Gram-negative *Shigella flexneri* and show that, in both cases, Mfd plays an important role in bacterial survival in the context of NO-induced stress. We provide insights into the role of Mfd during bacterial pathogenesis and into the mechanisms of DNA damage and repair after NO stress.

## Results

### Mfd is required for virulence and bacterial growth *in vivo*

*B. cereus* is an emerging pathogenic bacteria, which can infect hosts as diverse as insects and humans^23^,^24^,^25^,^26^,^27^. We used this remarkable property of wide host spectrum to develop a direct approach to identify new *B. cereus* virulence factors. A library of mutated genes of the *B. cereus* 407 (Bc 407) strain was constructed^28^ and over 1500 mutants were individually tested for their capacity to kill *Bombyx mori* insect larvae. A strain mutated in the *trcf* gene was identified as avirulent. This gene (Bc0058 in the *B. cereus* reference strain ATCC 14579) encodes for the Mfd protein.

**Figure 1:**
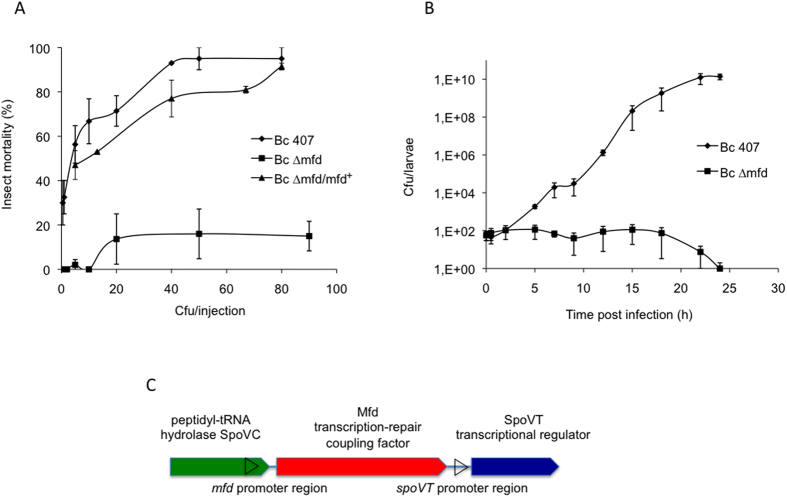
(**A**) Various doses of wild-type (Bc 407), *mfd* mutant (Bc ∆*mfd*) and the complemented Bc ∆*mfd/mfd*^+^ strains were injected into the hemocoel of *B. mori* larvae. Insect mortality was recorded 24 h post infection. The results are mean values of at least three independent experiments. (**B**) *B. mori* were infected with 50 cfu of *B. cereus* wild-type (Bc 407) or *mfd* mutant (Bc ∆*mfd*) strains. After the indicated times, larvae were crushed in PBS medium and cfu were counted by plating serial dilutions on agar plates. The results reported are mean values of at least five independent experiments. (**C**) *mfd* gene environment in *Bacilli* is schematically represented. The promoter regions as defined in http://genome.jouy.inra.fr/cgi-bin/seb/viewdetail.py?id=mfd_60430_63963_1 is indicated as black arrow.

A stable *mfd* deletion strain was constructed by insertion of a kanamycin-resistance cassette into the *mfd* gene. *B. cereus* growth of wild-type and *∆mfd* mutant strains did not differ in LB medium (Fig. S1). The pathogenicity of the wild type and mutant strains was tested in *B. mori* (Fig. 1A). Deletion of *mfd* induced a drastic loss of virulence compared to the wild-type strain. To confirm that the chromosomal deletion did not induce a polar effect, the *mfd* mutant strain was genetically complemented by introduction of a plasmid carrying a functional *mfd* gene and the corresponding promoter region. Complementation of the ∆*mfd* strain by the *mfd*^+^ gene restored the wild-type virulence phenotype (Fig. 1A). In addition, a plasmid carrying the *spoVT*^+^ gene situated downstream of *mfd* was also introduced into the ∆*mfd* mutant (Fig. 1C). The *spoVT*^+^ gene did not restore the virulence capacity of the mutant, which remained similar to that of the single ∆*mfd* mutant, showing that the downstream *spoVT* gene is not involved in the *mfd* mutant phenotype (Fig. S2). This finding in addition to the complement data shows that Mfd is required for *B. cereus* virulence in an insect model of infection.

We assessed the survival of the strains during infection by crushing the insects and dilution plating. In sharp contrast to the wild type stain, the *∆mfd* mutant was unable to grow *in vivo* and no bacteria were recovered 24 h after infection (Fig. 1B). Thus, Mfd is required for bacterial survival during the infectious process.

### Mfd is required for *B. cereus* resistance to NO stress produced *in vitro* or by phagocytic cells

**Figure 2:**
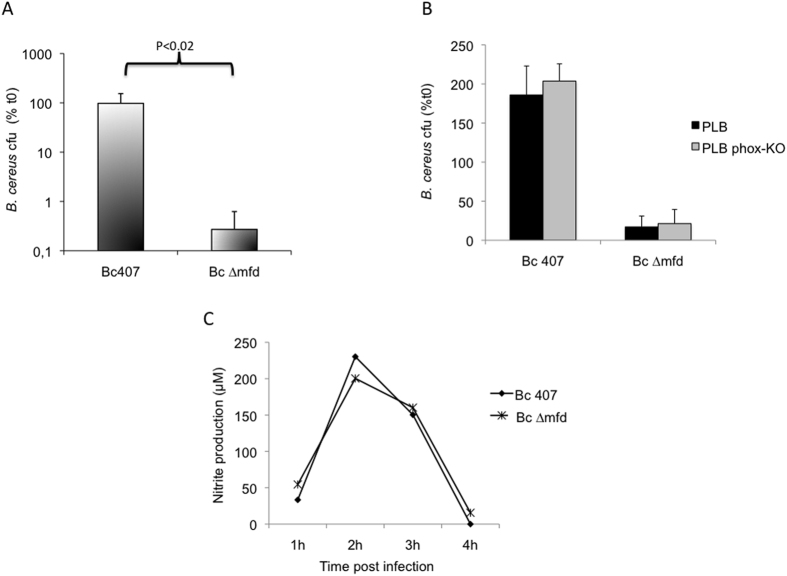
Human monocytic-like cells PLB (**A**) and PLB deficient for the ROS response (PLB phox-KO) (**B**) were incubated with *B. cereus* wild-type and *mfd* mutant for 4 h and bacterial survival was calculated by plating serial dilutions on agar plates. The cfu counts were normalized to the initial cfu (t0). The results reported are mean values of at least three independent experiments each in triplicate. P values are calculated using the Student test. (**C**) Nitrite concentration was measured in the PLB cell supernatant collected after each time of infection by the Griess method. This graph represents a set of representative data out of three independent experiments done in triplicate.

**Figure 3:**
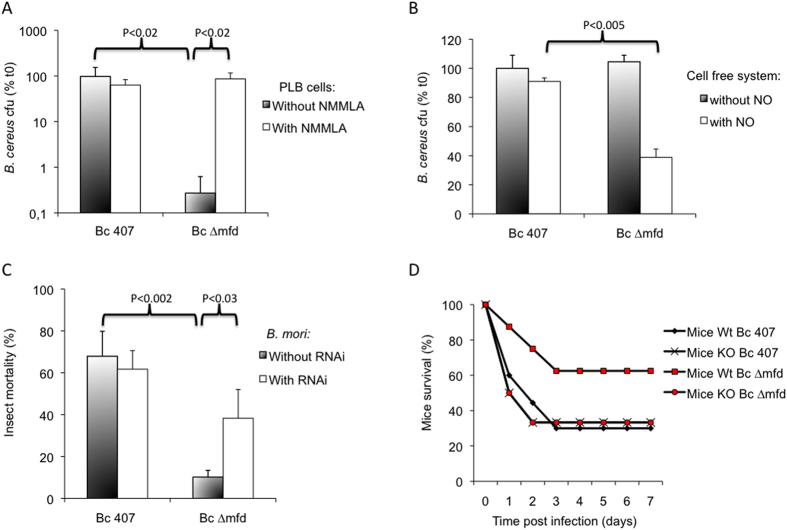
(**A**) Human monocytic-like cells, PLB were infected with *B. cereus* wild-type and the *mfd* mutant for 4 h in the presence or absence of 1 mM NMMLA (an NO inhibitor). Bacterial survival was calculated by plating serial dilutions on agar plates. Cfu counts were normalized to initial cfu. The results reported are mean values of at least three independent experiments each in triplicate. P values are calculated using the Student test. (**B**) Bacteria were exposed directly to chemically generated NO (500 μM sodium nitrite) for 4 h in a cell-free system. Bacteria were then harvested and plated on agar plates to evaluate bacterial survival. Cfu counts were normalized to initial cfu. The results reported are mean values of at least five independent experiments. P values are calculated using the Student test. (**C**) NO production in the insect *B. mori* was silenced by RNA interference (RNAi). Larvae injected with either 1μg of double-stranded NOS RNA (dsRNA) or water only (control) were infected with either wild-type *B. cereus* or the ∆*mfd* mutant and insect mortality was recorded after 24 h. (**D**) C57/Bl6/Sev129 mice (10 Mice wt) and NOS2−/− mice (10 KO Mice) were inoculated intranasally with *B. cereus* wild-type and ∆*mfd* mutant strains (5.10^6^ cfu/mice). Mortality was recorded daily for 7 days.

We tested whether the failure of the *∆mfd* mutant to survive in the insect host was due to its sensitivity to host cellular defenses. The human monocytic-like cell line PLB was infected with the two strains and bacterial survival was assessed (Fig. 2A). The wild-type strain survived in the PLB cells whereas the mutant did not (P < 0.02), implying that a cellular response was preventing growth of the mutant. Host defense against bacteria is predominantly mediated by cellular immune mechanisms. Phagocytic neutrophils and macrophages infiltrate inflamed areas, where they produce an extensive array of toxic substances. Among the secreted chemicals are oxygen (ROS) and nitrogen (NOS) species, which are known to induce DNA damage and mutations^3^. To test the effect of ROS on the *mfd* mutant growth, the wild type cell line PLB and its mutated counterpart deficient in ROS production (gp91phox-KO) were infected with the strains (Fig. 2B). The *B. cereus* wild type strain grew equally in both cell lines. The *mfd* mutant grew neither in the wild type nor in the ROS deficient cells, suggesting that suppression of ROS was not sufficient to restore growth of the *mfd* mutant. This is consistent with another finding showing that Mfd is not required for transcription recovery following oxidative stress in *E. coli*^29^. We measured similar nitrite production in the supernatant of cells infected with wild-type or mutant strains (Fig. 2C), suggesting that in both cases, infection of PLB cells induced a NO response. To test the role of Mfd during resistance to NO produced by cells, the survival rates of *∆mfd* and wild-type strains were assessed after incubation of PLB cells in the presence of an inhibitor of the nitrogen response, N^G^-monomethyl-L-arginine (NMMLA), a competitor of the iNOS enzyme (Fig. 3A). The ∆*mfd* mutant did not survive following incubation of cells in the absence of NMMLA. By contrast, in the presence of the inhibitor, the survival rates of *∆mfd* and wild-type strains were similar (P > 0.26) strongly suggesting that Mfd plays a role in NO resistance.

In a second type of experiment, bacteria were exposed directly to chemically generated acidified nitrite in a cell-free system (Fig. 3B). The wild-type strain survived, whereas survival of the ∆*mfd* mutant was impaired (P < 0.005). The concentration of NO able to reduce by 50% the survival of the strains (IC 50) was measured by dose-response experiments. The NO-IC 50 required for the wild type strain was of 4.12 ± 1.2 mM and was thus 2.5 times higher that the IC 50 for the *mfd* mutant, which was 1.61 ± 1.3 mM. These data implicate Mfd in the resistance to NO, a critical mediator of the host innate immune response. We tested the impact of other mutagenic agents on wild type and *mfd* mutant strain survival. The *mfd* mutant was not sensitive to UV or mitomycin treatments (not shown), consistent with previous reports showing in the ∆*mfd* mutant a high UV mutability despite a minimal UV or mitomycin sensitivity^30^,^31^.

**Figure 4:**
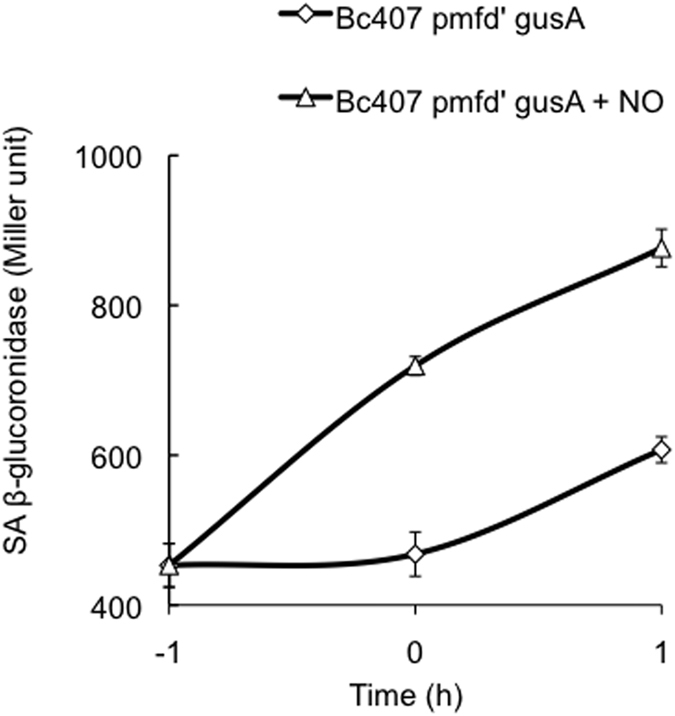
m*fd* gene expression was assessed by measuring the ß-glucoronidase activity of the Bc407 [pHT304G-p*mfd’gus*] strain grown in LB medium at 37 °C with or without NO. Samples were taken every hour from one hour before to one hour after the entry of the bacteria in the stationary growth phase. Specific activity is expressed in units of β-glucuronidase per milligram of protein (Miller units). The results reported are mean values of three independent experiments.

It has been previously reported that the expression of *mfd* in *B. subtilis* is upregulated after ethanol or H_2_O_2_ exposure (Basysbio, http://genome.jouy.inra.fr/cgi-bin/seb/index.py). We measured the impact of NO on *B. cereus mfd* gene regulation by measuring the β-glucoronidase activity of a transcriptional fusion between the *mfd* promoter and the *gus* gene (Fig. 4). The *mfd* promoter showed a constitutive activity that increased throughout bacterial growth in the absence of NO. In the presence of NO, *mfd* gene expression was significantly upregulated.

### Mfd is required for *B. cereus* resistance to the host immune NO response

To determine whether the *mfd* mutant growth was inversely correlated with the host NO response *in vivo*, we used two animal models: *B. mori* larvae and mice. NO production in insects was shut down by injection of NOS-siRNA into the insect hemocoel prior to infection with wild-type and *mfd* mutant strains. Inhibition of NO production had no significant effect on insect mortality after infection by the wild-type strain, indicating that this strain counteracts the NO host defense (Fig. 3C). In contrast, inhibition of NO production significantly increased the virulence of the *∆mfd* strain compared with insects producing an NO-response (P < 0.03). Thus, Mfd is essential for virulence in the context of NO-stress *in vivo*. The mortality induced by the *mfd* mutant in the context of NO inhibition was intermediate and did not reach the level of mortality induced by the wild type strain (P > 0.067) indicating that some other lesions, independent of NO, may also be dealt with by Mfd.

Wild-type and *∆mfd* strain virulence was also assessed in wild-type mice and in mice deficient for NO production (iNOS-KO) (Fig. 3D). The percentage of surviving wild-type mice infected with the *B. cereus* wild type strain decreased sharply following infection, while the survival of wild-type mice infected with the *∆mfd* mutant was significantly higher. These results show that Mfd makes a substantial contribution to *B. cereus* pathogenesis in a mammalian model of infection. The survival of iNOS-KO mice infected with wild type and *∆mfd* mutant strains was similar to the survival of the wild-type mice infected with the wild-type strain. Thus, in the absence of NO production, the *mfd* deletion had no effect on virulence. These findings imply that Mfd counteracts the deleterious effect of NO on bacteria *in vivo*.

Our study as a whole provides significant insights into the previously undescribed role of Mfd during bacterial pathogenesis and, in particular, demonstrates its involvement in the mechanisms of defense against NO stress *in vivo*.

### Mfd as a universal virulence factor implicated in NO stress resistance

**Figure 5:**
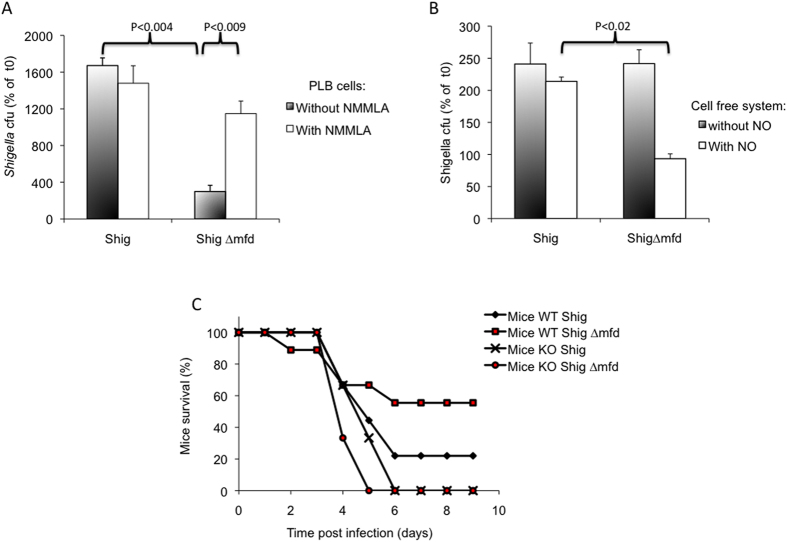
(**A**) PLB cells were infected with *S. flexneri* wild-type (Shig) and the *mfd* mutant (Shig ∆mfd) in the presence or absence of 1 mM NMMLA for 24 h. Bacterial survival was calculated by plating dilutions on LB agar plates. Cfu counts were normalized to initial cfu. The results reported are mean values of at least three independent experiments each in triplicate. P values are calculated using the Student test. (**B**) Bacteria were exposed to chemically generated NO (500 μM sodium nitrite) for 4 h in a cell-free system. Bacteria were harvested and plated on agar plates to evaluate bacterial survival. Cfu counts were normalized to initial cfu at t0. The results reported are mean values of at three independent experiments. P values are calculated using the Student test. (**C**) C57/Bl6/Sev 129 mice (9 Mice wt) and NOS2−/− mice (9 Mice KO) were inoculated intranasally with *S. flexneri* wild-type and ∆*mfd* mutant bacteria (10^7^ cfu/mice). Mortality was recorded daily for 9 days.

To determine whether *mfd* inactivation affects virulence in another bacterial species, we studied the effect of *mfd* deletion in *Shigella flexneri. S. flexneri* is a Gram-negative *Bacillus* and is the major etiological agent of dysentery in developing countries^32^,^33^. NO is produced following *S. flexneri* infection but does not result in clearance of *S. flexneri* from infected mice, suggesting that bacteria escape the NO response^34^. To study the role of Mfd in the resistance of *Shigella* to NO stress, we evaluated bacterial growth after incubation with phagocytic PLB cells, with and without the NO inhibitor NMMLA (Fig. 5A). In the absence of NMMLA, the wild-type strain was able to grow, whereas growth of the *Shigella ∆mfd* mutant was severely impaired (P < 0.004). Addition of the NO inhibitor increased the survival of the *∆mfd* mutant (P < 0.009) to a level similar to that of the wild-type strain (P > 0.13).

Survival of the *∆mfd* mutant was further tested in NO stress conditions in a cell-free system (Fig. 5B). The growth of the *Shigella ∆mfd* mutant in the presence of nitrite was much weaker than that of the wild-type strain (P < 0.02). The survival of the wild type and mutant *S. flexneri* strains in the cell free system without nitrite exposure was identical (Fig. 5B).

The role of Mfd in resistance to NO stress was also tested *in vivo* in wild type and iNOS-KO mice (Fig. 5C). Survival of wild-type mice infected with the *∆mfd* mutant was significantly higher than survival of these mice infected with the wild-type *Shigella* strain. Thus, Mfd is required for full *Shigella* virulence. In mice deficient for NO production, both wild type and *∆mfd* mutant strains were highly virulent, and mouse survival was even lower than for wild-type mice.

These experiments demonstrate that Mfd plays an important role in promoting bacterial survival in the context of NO-stress during the infection process of two very divergent bacteria.

### NO stress results in DNA damage in the ∆*mfd* mutant

The role of NO during infection clearance has been described in several bacteria and several molecular mechanisms involved in the repair of NO-induced DNA lesions have also been identified^7^,^8^,^9^,^35^,^36^. However, the involvement of Mfd during the repair of NO-induced lesions has never been reported. Mfd is involved in DNA repair, and we report that Mfd contributes to the resistance of *B. cereus* and *Shigella* to NO, therefore, Mfd may be required to repair DNA damage resulting from NO stress during infection. Pulse field gel electrophoresis (PFGE) has been widely used to detect the formation of linear chromosomal fragments. Indeed, cell lysis performed in the plugs ensures that only linear chromosomes enter the gels, while circular molecules remain in the wells^37^,^38^.

**Figure 6:**
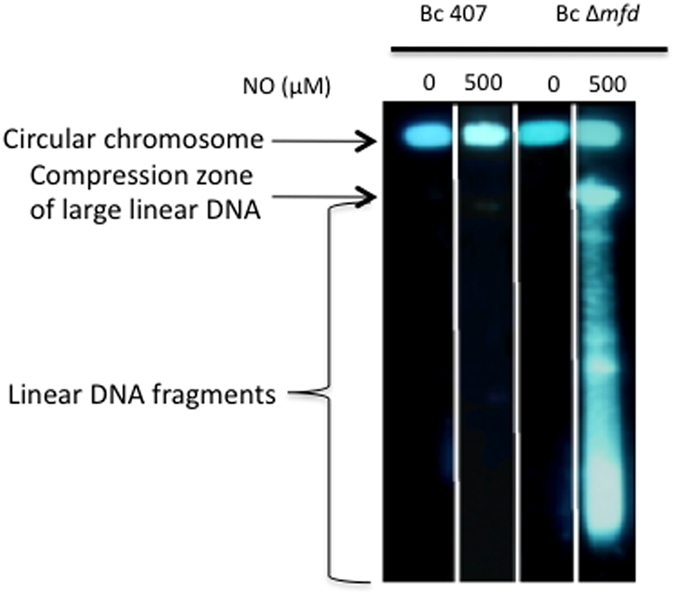
Chromosomal linear DNA was visualized in *B. cereus* by pulsed field gel electrophoresis. Wild-type and ∆*mfd* mutant strains were grown until mid exponential growth phase and treated with 500 μM NO in a cell free system. DNA corresponding to 10^8^ bacteria was incorporated in low melting agar gel plots and analyzed by pulsed field electrophoresis (PFGE). Circular chromosomal DNA are blocked in the loading well, whereas linear DNA migrate through the gel. DNA degradation is visible as a smear below the linear DNA band. This shows representative data out of 5 independent experiments.

In the absence of NO, linear DNA was not detected, neither in the wild type nor in the ∆*mfd* strains (Fig. 6). However, addition of NO to the ∆*mfd* mutant strain resulted in the appearance of linear DNA reflecting chromosome breakage. Moreover, NO treatment induced DNA degradation in the ∆*mfd* mutant, visualized as a smear during electrophoresis. Thus, the presence of NO induces DNA fragmentations and Mfd is required to prevent or repair them.

### Mfd helps to counteract the mutagenic effect of NO

No specific pattern of mutations has yet been attributed to NO stress. We aimed to identify the rate and type of mutations and the implication of Mfd in the repair of these DNA damages. To assess the rate and type of NO-induced mutations, we constructed *B. cereus* strains expressing *gusA* and measured the percentage of strains loosing their blue color on X-glucuramidase plates after sub-lethal *in vitro* NO treatment. In the presence of tetracycline, which ensures that all colonies carry a plasmid, no white colonies were found with the wild-type *B. cereus* strain, whereas in the ∆*mfd* mutant, 1±0.4 colonies per 2.8 × 10^3^ were white. The P_*aphA3*_-*gusA* region of the plasmids (including the promoter region and the *gusA* coding sequence) from 16 independent non-clonal colonies was sequenced. Three types of mutation were identified, all of them being transversions (G:C to T:A, A:T to C:G and C:G to G:C), mapping at four different sites of the cloned region (Table 1).

To ensure that the mutations were not generated during replication in *E. coli* we performed several controls, including the sequencing of plasmids issuing from blue colonies after NO treatment, and control plasmids from ∆*mfd* strains grown in LB medium without NO treatment. In both cases, we observed no mutation despite replication in *E. coli*.

Taken together our data show that in the absence of Mfd, NO stress causes at least three types of base substitutions that occur preferentially at specific positions.

## Discussion

This study reports the crucial role of the Mfd protein during pathogenesis of bacteria as different as *B. cereus* and *S. flexneri* in both invertebrate and mammal hosts. Despite the numerous publications addressing the importance of Mfd in DNA repair and adaptive mutagenesis, to our knowledge its importance during bacterial pathogenesis has not been previously reported.

We demonstrate that Mfd is essential for specific resistance to the deleterious effect of nitrogen stress imposed by host phagocytes. We observed that in *B. cereus*, in addition to its basal constitutive expression, *mfd* gene expression is upregulated in the presence of NO. This is in agreement with previous findings using a tiling DNA microarray covering the entire chromosome of *B. subtilis* and showing a multiple regulatory system of *mfd* gene expression (http://subtiwiki.uni-goettingen.de/wiki/index.php/Mfd): *mfd* expression is controlled by SigA and SigB, two regulators implicated in “house keeping” and stress-induced response, respectively^39^,^40^. A SigB-dependent regulation is in agreement with the higher expression of *mfd* in NO stress conditions.

Production of reactive nitrogen species, such as NO is an important component of the host immune defense against bacteria. NO has toxic effects on several molecules including nucleic acids, thereby inducing DNA damage and strand breaks. We demonstrate a link between NO-induced bacterial DNA damage and DNA repair by Mfd. The appearance of linear DNA during the PFGE experiments suggests that NO induces DNA double-strand breaks (DSBs) in the ∆*mfd* mutant^37^,^38^. DSBs are readily detected by PFGE in cells deficient for RecBCD-dependent homologous recombination, owing to the absence of DSB repair^38^. This is in agreement with previous findings showing the implication of RecBC in NO resistance of *Salmonella enterica*^9^. We hypothesize that Mfd could remove a RNAP arrested by a DNA lesion, or repair protein complexes, from the path of recombination-dependent replication forks after fork breakage due to replication blockage.

Previous findings on *Salmonella typhimurium* showed that NO induces DNA damage targeted by the Base Excision Repair (BER) pathway^8^. BER is involved in the recognition of modified bases by specific DNA glycosylases, which action on NO-induced DNA damage can induce homologous recombination^35^. As the formation of DSBs is usually linked to the activation of RecBC-dependent homologous recombination^38^, it would be interesting to study the link between Mfd, RecBC and BER following NO genotoxic bacterial DNA damage.

Absence of *mfd* reveals three types of mutation after NO treatment, all of them being transversions (G:C to T:A, A:T to C:G and C:G to G:C). G:C to T:A and A:T to C:G transversions are usually induced by loss of MutT, Fpg and MutY enzymes, which all deal with G oxidation. C:G to G:C probably also involves NO-induced G lesion^35^,^36^. Thus, NO treatment in a *mfd* mutant generates a high level of DNA fragmentation and increases point mutation frequency. These two phenomena might occur independently of each other, or RecBC-dependent DNA replication may be mutagenic in our conditions.

We speculate that the NO damages occur rapidly and throughout the bacterial chromosome. Indeed, *B. cereus* does not possess any pathogenicity island and we observed that the *mfd* mutant survival is completely impaired *in vivo*, suggesting a rapid and considerable extent of damage. Alternatively, Mfd may also have a general impact on virulence by indirectly controlling the production of virulence genes. For instance, in *Clostridium difficile*, Mfd controls the level of toxin gene expression maybe by relieving RNAP stalled at roadblocks created by the toxin repressors CodY and CcpA^41^.

Taken together, our findings demonstrate an important role of the DNA repair enzyme Mfd during bacterial resistance to NO stress, by limiting the genotoxic effects of NO generated by the host inflammatory response. These findings allow better defining the mechanistic pathway of bacterial resistance to NO and the efficient contribution of Mfd to bacterial survival during pathogenesis. Mfd is well conserved among bacterial species and these observations reveal a mechanism that may be used by a large spectrum of bacteria to overcome host immune responses, and in particular the damaging properties of reactive nitrogen species.

## Materials and Methods

### Ethics Statement

All experimental protocols were conducted in compliance with French legislation and approved by the author local ethic committee, named Comethea under the agreement number: B78–720. The experiments were performed by INRA-UIERP animal facilities in Jouy en Josas, France (N° SIRET INRA Jouy en Josas: 18 0070039 00078) in conformity with ethics regulations as established by EU legislation (2010/63/EU).

No samples were obtained from human patient for this study.

### Genomic mutant bank screening and identification of avirulent mutants

The *B. thuringiensis* strain 407 Cry^−^ (Bc 407) was used as a model for *B. cereus*. This strain has been cured of its plasmid, is acrystalliferous, and shows high phylogenic and phenotypic similarity with the *B. cereus* reference strain ATCC 14579 and is therefore considered as a *B. cereus* strain^42^. A random insertion mutagenesis library was constructed in Bc 407 by using the mini-Tn10 transposon as previously described^43^. Insertion mutants were screened for loss of virulence in *Bombyx mori* larvae, following their inoculation into the hemocoel^28^,^44^. Chromosomal DNA regions flanking the insertion loci were cloned, and their nucleotide sequences were determined as previously described^43^.

### Bacterial strain and mutant construction

The Bc 407 *∆mfd* mutant was constructed as follows. The *mfd* gene was disrupted through double homologous recombination using the thermosensitive vector pMAD. *Bam*HI-*Xba*I (515 bp) and *Pst*I-*EcoR*I (512 bp) DNA fragments corresponding to upstream and downstream regions of the *mfd* gene were generated from the Bc 407 chromosome by PCR using the primer pairs:

mfd-1 (5′-CGCGGATCCGTAGGCTCCATTAACGCAG-3′),

mfd-2 (5′-GCTCTAGACACCAAGTAACGCCACTAAATC-3′), and

mfd-3 (5′-AACTGCAGGAGGTGTTTCTGCAATTGAGG-3′),

mfd-4 (5′-CCGGAATTCGCTGCCTCATTTCTACTG-3′).

A Kan^R^ cassette carrying a *kan* gene was purified from pDG783^45^ as a 1.6 Kb *Xba*I-*Pst*I fragment. The amplified DNA fragments and the Kan^R^ cassette were digested with the appropriate enzymes and assembled together by ligation to produce a “*mfd*-upstream”-“Kan^R^ cassette”-“*mfd*-downstream” *Bam*HI-*EcoR*I fragment, which was then inserted between the *Bam*HI and *EcoR*I sites of pMAD. The resulting plasmid was introduced into Bc 407 by electroporation^46^ and the *mfd* gene was deleted by a double crossover event as previously described^47^. Chromosomal allele exchange was confirmed by PCR with oligonucleotide primers located upstream from mfd-1, mfd-5 (5′-AACTGCAGGCAGACACTGCGGAGG-3′) and downstream from mfd-4, mfd-6 (5′-TGCTCTAGACCTTCGGGATTACTACCCTGCC-3′), and in the Kan^R^ cassette (5′-CGGGTCGGTAATTGGGTTTG-3′), (5′-GCAGCTGCACCAGCCCCTTG-3′). The insertion mutant strain was designated Bc 407 ∆*mfd*.

To complement the Bc 407 *∆mfd* mutant, the *mfd* gene, including the coding sequence and the promoter region (Fig. 1C and http://subtiwiki.uni-goettingen.de/wiki/index.php), was obtained using the primer pair mfd-5 and mfd-6, and the sequence of the obtained fragment was verified. The pHT315 cloning vector^46^ containing the complete *mfd* gene and its promoter was used to transform Bc 407 Δ*mfd* strain by electroporation. Transformants were selected for resistance to erythromycin. The resulting new strain was designated Bc 407 Δ*mfd*/*mfd*^+^.

In *Bacilli*, the *mfd* gene is located between a gene coding for a peptidyl tRNA hydrolase and a gene coding for an AbrB-like transcription regulator (spoVT). The *spoVT* gene situated immediately downstream of *mfd* was amplified using the primers (5′-AACTGCAGGAGGTGTTTCTGCAATTGAGG-3′), and (5′TGCTCTAGACCCCACGCCAAAAGGCTTG-3′). This fragment contained the *spoVT* gene with its own promoter as defined in http://subtiwiki.uni-goettingen.de/wiki/index.php, and was inserted into the *Pst*I and *Xba*I sites of the pHT315 cloning vector. The pHT315 vector containing the *spoVT* gene with its own promoter was introduced into the ∆*mfd* strain by electroporation. This new strain was designated Bc 407 Δ*mfd*/*spoVT*^+^.

Transcriptional *mfd*′*gusA* fusion was constructed using DNA fragments corresponding to the promoter region of *mfd* generated by PCR using the primer pair pmfd-1 (5′-AGAAACGCTCGCATCTTATCCTG-3′) and pmfd-2 (5′-AGACGTTGCCATCCCTGATATAAG-3′). The promoter region was then inserted in the pHT304-18GusA plasmid^48^. The recombinant plasmid was introduced into Bc 407 by electroporation. Transformant was named Bc407 [pHT304G-p*mfd’gus*].

Strains Bc 407 {pHT1618′ P_*aphA3*_-*gusA*} and Bc 407 ∆*mfd* {pHT1618′ P_*aphA3*_-*gusA*} were constructed as follows. To construct pHT1618′ P_*aphA3*_-*gusA*, the constitutive promoter of the Kan^R^ cassette (P_*aphA3*_) was amplified as a 571 bp *Pst*I-*Xba*I fragment from pDG783 using the primers papha3-1 (5′-GCATGCCGTCAGGTGATAAACC-3′) and papha3-2 (5′-GCTCTAGACAATTCCGGTGATATTCTCATTTTAGCC-3′), and the *gusA* gene was amplified by PCR from pTUM177^49^ with primers GUS1 (5′-TGCTCTAGATAAAGGAGAAAATTTTATGTTACGTCC-3′) and GUS2 (5′-CCGGAATTCGGTGCGCCAGGAGAGTTG-3′) as a *Xba*I-*Eco*RI fragment. These fragments were inserted between the *Pst*I and *Eco*RI sites of pHT1618, which was used to transform the strains Bc 407 and Bc 407 ∆*mfd*.

The *Shigella flexneri* 2a M90T strain was used as the wild-type reference strain. A ∆*mfd* mutant of this strain was constructed as follows. The *mfd* gene (S1198) was interrupted by double recombination as previously described^50^. Upstream and downstream regions of the *mfd* gene were generated from the *S. flexneri* 2a M90T chromosome by PCR using the primer pairs: S-mfd-1 (5′-GCTCTAGAGCGCTCCACCAGCCTGCTG-3′) and S-mfd-2 (5′-CGGGGTACCCCGCGCGTGGCGTATTCGCCG-3′), and S-mfd-3 (5′-CGCGGATCCCGGCCTGCTGCCAGATCCGGC-3′) and S-mfd-4 (5′-CCGGAATTCCGGGCGCGGATGTTTGCCG-3′). A Kan^R^ cassette carrying a *kan* gene was excised from pUC18 K2 as a 839 bp fragment. The DNA fragments were digested with the appropriate enzymes and inserted into pGP704^50^. The resulting plasmid was introduced into M90T by electroporation and the *mfd* gene was deleted by a double crossover event. Chromosomal allele exchange was confirmed by PCR with oligonucleotide primers located upstream from S-mfd-1, S-mfd-5 (5′-CCCAAGCTTAAGAGGTGCCGTTGCGCCGCC-3′) and downstream from S-mfd-4, S-mfd-6 (5′-TCCCCCGGGGGGTTATCCGGTCCAGCCGGC-3′), and in the Kan^R^ cassette (5′-GCAAGGCGATTAAGTTGGGTAACGCCAGGG-3′), (5′-CCCCGCGCGTTGGCCGATTCATTAATGCAG-3′). The insertion mutant strain was designated *S. flexneri* ∆*mfd*. Growth curves of wild type and mutant strain were similar in LB or RPMI medium at 37 °C.

### NO inducer and inhibitor

Bacteria were exposed directly to chemically generated NO + in a cell-free system as described by Miyagi *et al*.^51^ with modifications. Briefly, bacteria were grown in LB medium until mid exponential growth phase at 37 °C with shaking. Cultures were then diluted to 10^4^ cfu/ml in RPMI-1640 medium (Invitrogen) at acidic pH (pH 5.4) in the presence of 0 to 10 mM sodium nitrite (Walco Pure Chemical Industries Ltd); under these acidic conditions, NO+ is generated from the sodium nitrite by a chemical reaction^51^. The bacteria were then cultured at 37 °C for 0 to 4 h, harvested and plated on agar plates to evaluate bacterial survival. Using this cell free assay, the NO+ concentration required to decrease by 50% the survival rate of each strain (IC_50_) was calculated using the GraphPad PRISM software (version 6.0, GraphPad Software, San Diego, CA) with non-linear regression. Data are means of at least 5 independent experiments.

To inhibit the nitrogen response in cellular assay, the NO inhibitor NMMLA N^G^-monomethyl-L-arginine (Sigma) was used at 1 mM. NMMLA competes with L-arginine for the site of action of the iNOS enzyme.

### UV and mitomycin treatment

*B. cereus* wild type and mutant strains were grown at 37 °C under agitation until mid exponential growth phase and diluted to 5. 10^7^ cfu/ml. Serial dilutions were plated as spots of 10 μl on agar plates and exposed to UV light (5J/m2) for 0 to 15 seconds. Alternatively, serial dilutions were plated on agar plates containing 50 ng/mL mitomycin C. Plates were incubated ON at 37 °C and bacterial survival was assessed by observing the size of the spot colonies on the plates for each dilution.

### β-glucuronidase assays

The Bc407 [pHT304G-p*mfd’gus*] was grown in LB medium, pH 5.4 at 37 °C until one hour before the bacterial entry into stationary phase (t-1). At t-1, 0.4 mM NO was added to the culture and samples were taken every hour from one hour before to one hour after the entry of the bacteria in the stationary growth phase. Determination of β-glucoronidase activity was achieved as previously described^48^. Specific activities are expressed in units of β-glucuronidase per milligram of protein (Miller units).

### Cell culture and infection

The human monocytic-like cells, PLB-985 and PLB deficient for Gp91phox, one of the multicomponents of the enzyme NADPH oxidase responsible for ROS production (gp91phox−/−), were used (kindly provided by Dr. Benaroch, Institut Curie, Paris, originating from M. Dinauer laboratory^52^). Cells were maintained in RPMI-1640 medium supplemented with 10% fetal bovine serum (FBS, Invitrogen). Cells were incubated at 37 °C under a 5% CO_2_ atmosphere and saturating humidity. The day before use, cells were counted with a hematocytometer and seeded at a density of 3.10^5^ cells per well into multiwell disposable trays. Cells were infected with bacteria at a multiplicity of infection of 10 at 37 °C. After 4 h or 24 h bacteria and cells were removed from the flask and bacteria were counted by plating serial dilutions on agar plates.

Nitrite concentrations were measured by the Griess method: supernatant collected after each time of infection tested, and a standard, were mixed with an equal volume of Griess reagent (Sigma) and incubated for 10 min in the dark. The absorbance was then measured at 550 nm. The standards were obtained by serial dilution of NO.

### Insects and *in vivo* experiments

*Bombyx mori* larvae were infected by injection into the hemolymph as described elsewhere^44^,^53^. Groups of 20 last-instar larvae (same weight) were injected at the base of their last proleg with 10 μL of suspensions containing various doses of vegetative bacteria. Insect mortality was recorded after 24 h at 25 °C. To estimate the number of bacteria in living or dead larvae, the insect larvae were crushed and homogenized in sterile water; dilutions were plated onto LB agar plates^54^,^55^.

### RNAi in insects

NO production can be silenced at the transcription level by using RNA interference (RNAi)^56^. The silencing of gene expression by double-stranded RNA molecules is very efficient in *B. mori*^57^, and the gene coding for the inducible nitric oxide synthase-like protein (iNOS-LP) is present in only a single copy (in contrast to other invertebrate immune mechanisms for which many genes are present in several copies). Thus, RNAi is a powerful tool for manipulating NO levels in infected *B. mori*^58^. Double-stranded RNA (dsRNA) was produced from genomic DNA of *B. mori* using the primers 5′-ATTATGCTGAGTGATATCCCTCGAAGTTCTCGTCGTGAGCTA-3′ and 5′-TAATACGACTCACTATAGGGGAGAACCTCAGGAAGATGGATC-3′ and the Megascript RNAi Kit (Ambion). Larvae injected with either 1 μg of double-stranded NOS RNA (dsRNA) or water only (control) were infected with either wild-type Bc 407 or the *∆mfd* mutant (50 cfu/larvae) and mortality was recorded after 24 h.

### Mouse experiments

Mice experiments were performed by INRA-UIERP animal facilities in Jouy en Josas, France. C57/BL6/Sev129 mice, aged 6- to 8-weeks old were used for infections. Wild-type mice were obtained from Elevage Janvier, France. Mice with a targeted disruption of the NOS-2 gene, generated as previously described^59^ were generously supplied by Drs. J. D. MacMicking, C. Nathan (Cornell University Medical College, New York) and Jean Claude Jeanny, Institut des Cordeliers, animalerie centrale de la faculté de Pharmacie de Paris (health monitoring report from Harlan UK Technical service). PCR with DNA from tail biopsies was used for genotyping to verify the presence or absence of the NOS-2 gene^60^. Groups of 9 to 10 mice were inoculated intranasally with wild type or mutant bacteria (5.10^6^cfu/mouse for *B. cereus*, and 10^7^cfu/mouse for *Shigella* strains) as described in^34^,^61^. Mortality was recorded daily for 7–9 days.

### Pulsed-field gel electrophoresis (PFGE)

DNA damage was evaluated by pulsed field electrophoresis as described^62^ with slight modifications. Bc 407 and Bc 407 ∆*mfd* strains were grown until mid exponential growth phase at 37 °C with agitation. Bacteria where then submitted to NO stress in the cell free system with 500 μM NO for 4 h at 37 °C. For each condition, 10^8^ bacteria were harvested by centrifugation, incorporated into low-melting agar gel plugs, and lyzed. The DNA released was analyzed by electrophoresis in 1% agarose gels in 0.5X TBE at 6 V/cm for 24 h, with a linear pulse ramp of 3 to 52 s and a switching angle of 120°. Cell lysis performed in the plugs ensures that only linear chromosomes enter the gels, while circular molecules remain in the wells. All large linear fragments (usually >3 Mb) are comprised in the compression zone and thus appear as a singe band. DNA fragmentation and degradation appears as several bands or smear below this single band of linear DNA^63^.

### Mutation rate and type

Bacterial strains were grown until mid exponential growth phase at 37 °C with agitation. Bacteria where then submitted to sublethal NO treatment (125 μM) for 2 h at 37 °C. To assess the rate and type of mutations caused by NO, we constructed *B. cereus* strains expressing *gusA*: Bc 407 {pHT1618′P_*aphA3*_-*gusA*} and Bc 407 *∆mfd* {pHT1618’P_*aphA3*_-*gusA*}. These strains grow as blue colonies on LB-X-glucuramidase plates. The rate of non-neutral mutations specifically in the P_*aphA3*_-*gusA* gene was measured by scoring the appearance of white colonies on LB X-gluc plates supplemented with tetracycline (10 μg/mL) to maintain the pHT1618-derived plasmid. The pHT1618′ P_*aphA3*_-*gusA* plasmid was isolated from the white *B. cereus* ∆*mfd* colonies, and used to transform *E. coli* TG1. The plasmid was isolated from the *E. coli* transformants, and the P_*aphA3*_-*gusA* region was sequenced to identify the mutations obtained. To verify that the mutations observed led to a non-functional P_*aphA3*_-*gusA* gene, several plasmids isolated from white colonies were used to transform *E. coli*, re-isolated from *E. coli* and reintroduced into the Bc ∆*mfd* strain. In all cases, the plasmids conferred a white phenotype on LB X-gluc plates as expected (not shown). The original pHT1618′ P_*aphA3*_-*gusA* plasmid was used as control, and conferred a blue phenotype on LB X-gluc plates as expected.

## Additional Information

**How to cite this article**: Guillemet, E. *et al*. The bacterial DNA repair protein Mfd confers resistance to the host nitrogen immune response. *Sci. Rep.* **6**, 29349; doi: 10.1038/srep29349 (2016).

## Supplementary Material

Supplementary Information

## Figures and Tables

**Table 1:** Bc 407 {pHT1618’P_*aphA3*_-*gusA*} and Bc 407 *∆mfd* {pHT1618’P_*aphA3*_-*gusA*} were subjected to sublethal NO treatment (125 μM) for 2 h, then plated on LB-X-glucuramidase plates supplemented with tetracycline (10 μg/mL).

Clone number	Position#	Transition+	Amino acid change in the GUS protein*
1	643	G:C to T:A	Gly to Val
2	643	G:C to T:A	Gly to Val
3	1646	C:G to G:C	Tyr to Stop
4	1646	C:G to G:C	Tyr to Stop
5	1646	C:G to G:C	Tyr to Stop
6	1646	C:G to G:C	Tyr to Stop
7	1646	C:G to G:C	Tyr to Stop
8	1646	C:G to G:C	Tyr to Stop
9	1646	C:G to G:C	Tyr to Stop
10	1646	C:G to G:C	Tyr to Stop
11	1651	A:T to C:G	Tyr to Ser
12	1651	A:T to C:G	Tyr to Ser
13	1651	A:T to C:G	Tyr to Ser
14	1651	A:T to C:G	Tyr to Ser
15	2377	G:C to T:A	Arg to Leu
16	2377	G:C to T:A	Arg to Leu
Control plasmid		No mutation	
Control plasmidfrom ∆*mfd* in LB		No mutation	
Control bluecolonies		No mutation	

^#^Base position on the *gus* gene.

^+^Change in base pair compared to non mutated gene.

^*^Corresponding change in amino acid in the Gus protein.

The pHT1618’P_*aphA3*_-*gusA* plasmid was isolated from white colonies (only the *∆mfd* {pHT1618′P_*aphA3*_-*gusA*} strain gave white colonies), used to transform *E. coli* TG1, and isolated from the *E. coli* transformants; the P_*aphA3*_-*gusA* region was then sequenced. Position of mutations (from 1 to 2537 bp, corresponding to the sequenced fragment), base pair changes and the corresponding amino acid changes are indicated for each sequenced region. As controls, the corresponding regions in the initial plasmid, in the plasmid isolated from the *∆mfd* {pHT1618′P_*aphA3*_-*gusA*} strain grown for 2 h in LB medium, and in plasmids isolated from blue colonies after NO treatment were also sequenced.
