# Supplementary material for: The bacterial DNA repair protein Mfd confers resistance to the host nitrogen immune response
Source: Sci Rep. 2016 Jul 20;6:29349. doi: 10.1038/srep29349 (PMC4951645; doi:10.1038/srep29349)
Supplement: Supplementary Information [file srep29349-s1.pdf]

The bacterial DNA repair protein Mfd confers resistance to the host nitrogen immune response

Elisabeth Guillemet[1], Alain Leréec[1], Seav-Ly Tran[1@], Corinne Royer[2], Isabelle Barbosa[1#], Philippe Sansonetti[3], Didier Lereclus[1] and Nalini Ramarao[1*]

[1]Micalis Institute, INRA, AgroParisTech, Université Paris-Saclay, 78350 Jouy-en-Josas, France

[2]INRA, UMR 203, 11 Av J. Capelle, 69621 Villeurbanne, France

[3]INSERM U786, Institut Pasteur, 28 rue du Dr Roux, 75724 Paris Cedex 15, France

[@]Present address: Institute of Food Research, Norwich Research Park, and Norwich Medical School, University of East Anglia, NR4 7UA-7TJ, Norwich, UK

[#] Present address: ENS, UMR81, Paris, France

[*]Correspondence should be addressed to: Nalini Rama Rao, INRA, Unité MICALIS UMR-1319, Domaine de Vilvert, 78350 Jouy en Josas, France.

Tel: 00 33 1 34 65 28 63. E-mail: nalini.ramarao@jouy.inra.fr

Fig S1. *B. cereus* wild type and Δ*mfd* mutant strains were inoculated in LB medium at a starting optical density (OD) of 0.07 and grown at 25°C with agitation. The OD was measured every hour at 600 nm. This graph represents representative growth curves out of at least five independent experiments.

Fig S2. 100 cfu of *B. cereus* wild-type (Bc 407), Δ*mfd* mutant (Δ*mfd*), the Δ*mfd* mutant complemented with *mfd* (Bc Δ*mfd* / *mfd*$^{+}$) and the Δ*mfd* mutant harboring a plasmid carrying the *spoVT* gene (Δ*mfd*/*spoVT*$^{+}$) were injected into the hemocoel of *B. mori* larvae. Insect mortality was recorded 24 h post infection. The results are mean values of at least three independent experiments.

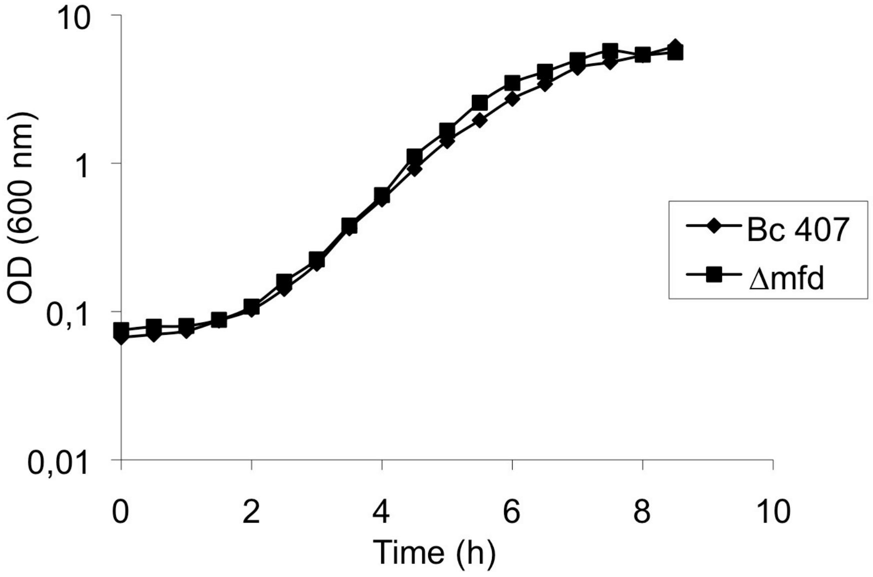

Figure S1

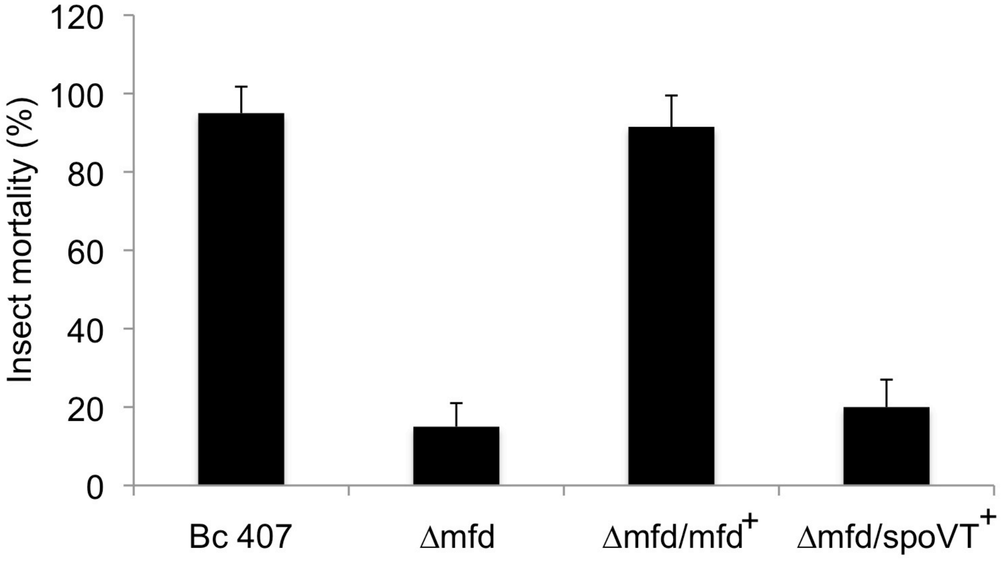

Figure S2
